# Transcriptome Changes Associated with Delayed Flower Senescence on Transgenic Petunia by Inducing Expression of *etr1-1*, a Mutant Ethylene Receptor

**DOI:** 10.1371/journal.pone.0065800

**Published:** 2013-07-09

**Authors:** Hong Wang, Genevieve Stier, Jing Lin, Gang Liu, Zhen Zhang, Youhong Chang, Michael S. Reid, Cai-Zhong Jiang

**Affiliations:** 1 Institute of Horticulture, Jiangsu Academy of Agricultural Sciences, Nanjing, China; 2 Department of Plant Sciences, University of California Davis, Davis, California, United States of America; 3 College of Horticulture, Nanjing Agricultural University, Nanjing, China; 4 Crops Pathology and Genetics Research Unit, United States Department of Agriculture, Agricultural Research Service, Davis, California, United States of America; University of Houston, United States of America

## Abstract

Flowers of ethylene-sensitive ornamental plants transformed with *ethylene-insensitive 1-1*(*etr1-1*), a mutant ethylene receptor first isolated from *Arabidopsis*, are known to have longer shelf lives. We have generated petunia plants in which the *etr1-1* gene was over-expressed under the control of a chemically-inducible promoter, which would allow expression of *etr1-1* to be initiated at the desired time and stage of development. Here, we showed that transgenic plants grew and developed normally without a chemical inducer. Semi-quantitative RT-PCR demonstrated that the abundance of transcripts of *Arabidopsis etr1-1* gene was substantially induced in flowers with 30 μM dexamethasone (DEX). Consequently, t he life of the flowers was almost doubled and the peak of ethylene production was delayed. We compared gene expression changes of petals with DEX to those without DEX at 24 h and 48 h by microarray. Our results indicated that transcripts of many putative genes encoding transcription factors were down-regulated by *etr1-1* induced expression at the early stage. In addition, putative genes involved in gibberellin biosynthesis, response to jasmonic acid/gibberellins stimulus, cell wall modification, ethylene biosynthesis, and cell death were down-regulated associating with *etr1-1* induced expression. We investigated time-course gene expression profiles and found two profiles which displayed totally opposite expression patterns under these two treatments. In these profiles, ‘the regulation of transcription’ was predominant in GO categories. Taking all results together, we concluded those transcription factors down-regulated at early stage might exert a major role in regulating the senescence process which were consequently characterized by cell wall modification and cell death.

## Introduction

Petal senescence is a complex programmed event which includes mineral transport and remobilisation, sugar synthesis and transport, synthesis and degradation of nucleic acids, amino acids, lipids, proteins and cell death compounds. This process is regulated by transcription factors, sugar and hormones [1]. For many ethylene-sensitive species, such as petunia, petal senescence is controlled and hastened by ethylene [2]. Discovering and achieving a mutant of the ethylene receptor is a significant step forward in genetic control of the postharvest flower quality. The ethylene receptor, ETR1, is a key part of the ethylene recognition and response pathway. In the absence of ethylene, the receptor works to suppress the expression of ethylene response genes. The mutant receptor *etr1-1,* which was originally identified in *Arabidopsis,* has been demonstrated to be unable to bind ethylene [3]–[6]. It remains constitutively active in its repression of ethylene response genes and therefore causes a dominant ethylene-insensitive phenotype. This mutant receptor has been successfully used in many species to confer ethylene insensitivity [7]–[10]. Flowers from these plants exhibit a reduction in ethylene sensitivity, resulting in an extension of the vase life. However, the reason why flowers of ethylene receptor mutant plants display extended longevity is not known.

Many of these studies used the 35S promoter to drive expression of *etr1-1* [11]–[14]. However, since ethylene signalling is involved in many other developmental processes, the use of the 35S promoter leads to other undesirable physiological effects, including problems with germination [12], [14], root formation [11], root development of cuttings [13], seed weight [12] and pathogen susceptibility [15]. A flower specific FBP1 promoter has recently been used to drive *etr1-1* expression in *Kalanchoe* [9] and *Campanula* [10] thereby producing longer-life flowers without impacting other developmental events [9]. Although effective, this approach has limited flexibility and precluded investigation into the timing of ethylene responses in flowers and the impact of ethylene signalling on other processes.

On the basis of the advantage of the precise timing and control of gene expression, several chemically inducible systems have been developed [16]. One of the chemically-regulated gene expression systems which have been described and successfully used is GVG expression system [17]. The system is activated by glucocorticoids such as dexamethasone, providing the ability to control tightly when and in which tissues expression is initiated [17].

This research tested the utility of using the inducible system for the control of *etr1-1* expression and the regulation of flower longevity. Based on an invaluable tool for studying ethylene response, cDNA microarray was used to investigate time-course gene expression changes due to induced ethylene-insensitive characteristics. The results shown that a large number of transcription factors were down-regulated at early stages in flowers with induced expression of *etr1-1*. Genes involved in gibberellin biosynthesis, response to jasmonic acid/gibberellins stimulus, cell wall modification were down-regulated at 24 h, however, genes encoding ethylene biosynthesis, vacuolar processing enzyme (VPE) and cell death were down-regulated at 48 h after *etr1-1* induced expression. Taken together, the results suggest that down-regulated transcription factors at early stage might play a crucial role in regulating the process of senescence on petunia with *etr1-1* induced expression.

## Materials and Methods

### Construction of plasmids and plant transformation

Primers for amplification of the *etr1-1* gene were designed from the known *Arabidopsis* sequence (5′- TAAACCCAACCAATTTTGACTTGAA-3′ and 5′-GGGTACTGTACGAGGG CATGTAA-3′). The fragments were amplified from *Arabidopsis thaliana* (Columbia ecotype, CS237) DNA. Sequences were confirmed by the U.C. Davis sequencing facility. The 3.2 kb fragment was cloned into the pTA7001 vector [17] in the sense orientation using the SpeI and XhoI sites. The vector was treated with Klenow after SpeI digestion but before XhoI digestions so that one end of the fragment was cloned into the vector using blunt-end cloning. This construct was transformed into *Agrobacterium tumefaciens* strain LBA4404 using electroporation [18]. PCR amplification was performed to further confirm the destination vector integrated with *etr1-1* gene.

The transformation was performed by U.C. Davis plant transformation facility. Briefly, bacteria were cultured overnight at 28°C in LB medium (10 g l^−1^ Bacto-peptone, 5 g l^−1^ Bacto-yeast extract, 10g l^−1^ NaCl, pH 7.2) (Difco, Detroit, MI) containing 50mg l^−1^ kanamycin (Sigma, USA). The bacterial culture for inoculation of explants was centrifuged and was diluted to 1∶200 in Murashige and Skoog (MS) salts and vitamins medium (4.4g l^−1^) (Sigma, USA). When the plants are 10–15 cm high, leaves of 3–8 from the top of wild-type *Petunia×hybrid* cv. ‘Mitchell diploid’ were sterilized and infected with bacterial cell suspension. Infected explants were placed on co-cultivation medium at 25°C for 2–3 days. After co-cultivation, these explants were transferred to a fresh regeneration and selection medium (4.4 g l^−1^ MS, 30 g l^−1^ sucrose, 2 mg l^−1^ 6-BAP, 0.01 mg l^−1^ NAA, pH 5.8) (Sigma, USA). Leaf discs inoculated with “empty” pTA7001 vector (without *etr1-1* gene) on regeneration medium with selective agent were designed as negative controls.

### Confirmation of the transformant

Transformants were transferred into soil (Metro-Mix 200, Sun Gro, Bellevue, WA, USA) and grown under artificial lighting (∼40 μM m^−2^ s^−1^, 16 h photoperiod, 25°C). The stigma of each flower was artificially pollinated and the flower without petals was enveloped with tape to avoid cross-pollination. T1 seeds were collected and sown on MS (Sigma, USA) medium with 20 mg ml^−1^ hygromycin (Sigma, USA) for the further selection of homozygous lines. The following procedure was performed: T1 seeds were first surface-sterilised in 15% bleach (Clorox, USA) with 0.01% Tween-20 (Sigma, USA) for 20 min and then 70% ethanol for 45sec, followed by three washes with sterile distilled water. The plates (20 seeds/plate) were kept at room temperature under continuous low fluorescence light (∼40 μM m^−2^ s^−1^) for 21 days. T1 seeds which displayed a 3∶1 ratio of survival on hygromycin medium were retained and moved to pots for harvesting T2 seeds. One hundred seeds from each line were germinated again on MS medium containing 20mg ml^−1^ hygromycin, and lines having 100% survival were identified as homozygous and used for further experiments.

PCR amplification was performed for further identifying transgenic lines. Genomic DNA of the petunia transformed *Arabidopsis etr1-1* gene was extracted using a hexadecyltrimethylammonium bromide (CTAB) method, as described previously [19]. PCRs were carried out in a total reaction volume of 20 µl, according to the procedure as the following: 5 min at 95°C followed by 40 cycles of 30 s at 94°C, 30 s at 56°C, and 1min at 72°C. Primer pairs: 5′-CCATCACACTAAATCTTGCACCA-3′ and reverse 5′- TTCGGTATGCCCGACTGTTTAG- 3′ were used. 1.0% agarose gel electrophoresis was performed using standard protocols.

### Plant growth conditions

Seeds of wild-type and T3 of two transgenic lines named E7H and E9G were germinated on 1/2 MS medium. The young seedlings were moved to 10 cm pots after 4 weeks. The pots were established in the greenhouse at the University of California, Davis. Plants were fertilised twice a week with N at 300 mg L^−1^ from 15N-5P-15K Cal Mag (Peters soluble fertiliser, The Scotts Co., Marysville, OH). Tap water was used for all other irrigations. During this period, the difference of seed germination and plant growth and development between transgenic lines and wild-type plants was monitored.

### Triple response assay

Surface-sterilized seeds of wild-type and T3 Seeds from transgenic line E7H were sown on MS plates with 30 μM dexamethasone at 25°C for 5 days in the dark. The seedlings were transferred to fresh MS plates in the presence or the absence of 20 μM ACC. After 3 days, hypocotyl length of the seedlings was measured. Three biological replicates were performed.

### Flower longevity

Flowers of lines E7H and E9G were harvested for vase-life evaluation when flowers were fully open but before the anthers had dehisced. Flowers were then placed in 2 ml tubes containing vase solution (Chrysal, USA) with 30 μM dexamethasone (Sigma, USA) or vase solution with 0.1% (v/v) DMSO (Sigma, USA) as a control. Flowers were monitored daily and were considered as senesced at the stage where the corolla exhibited loss of turgor (wilting) and the edges began to collapse. Flowers of wild-type were performed by similar treatment and used as control.

Dexamethasone (Sigma, USA) was stored as 10 mM solution in dimethyl sulphoxide (DMSO) at −20°C. Dexamethasone was added to vase solution to achieve induction in detached flowers. Unless otherwise stated, 30 μM dexamethasone or 0.1% (v/v) DMSO was used [20].

### Photoacoustic measurements of ethylene production

Ethylene emission was monitored in real-time by using a commercial laser-based ethylene detector (type ETD-300, Sense B·V., Nijmegen, The Netherlands) in combination with a gas handling system (type VC-6, Sensor Sense B·V.). Six samples per experiment were measured according to previously described methods [21], [22]

Flowers (about 330 mg fresh weight) of wild-type and E7H and E9G were placed into 250 ml volume closed glass cuvettes and continuously flushed with air at a constant flow of 3L h^−1^. During the period of the ethylene measurement, the cuvettes were kept at a constant temperature, 22°C. Ethylene emission from each sample was alternatively monitored by the ETD-300 for 15 min for each cuvette. In this study, six glass cuvettes with 6 ml H_2_O including 0.1% (v/v) DMSO were used per experiment. Two of them containing transgenic flowers were treated with 30 μM dexamethasone. Other two with transgenic flowers were used as control without the inducer of 30 μM dexamethasone. The fifth cuvette contained a wild-type petunia flower. A cuvette containing only H_2_O with 0.1% (v/v) DMSO was used to monitor the baseline. To remove any traces of external ethylene or other hydrocarbons, the air flow was passed through a platinum-based catalyser before entering the cuvettes. A scrubber with KOH and CaCl_2_ was placed before the ETD-300 to reduce the CO_2_ and the water content in the gas flow, respectively. For a better overview, the ethylene emission rate is displayed every 1 h. Each experiment was repeated 3 times with similar results.

### Molecular analysis of transgenic plants

#### Plant materials and RNA purification

Flowers of selected lines were harvested at the usual stage and placed in the dexamethasone or control solutions for gene expression and microarray analysis. The floral petals were harvested at 0, 24 h and 48 h after treatment, frozen in liquid nitrogen and stored at −80°C. Three independent experiments were carried out for each condition. Each biological replicate consisted of at least 5 different flower petals. RNA was extracted from mixed petals using the Trizol method (Invitrogen, USA), combined with Ambion RiboPure™ Kit (Ambion, USA). Briefly, samples were treated by Trizol method until the two phases came out after adding the chloroform and centrifuging. The upper phase was taken and an equal volume of 64% ethanol was added. DNA was removed from pellet with the Turbo DNA-free kit (Applied Biosystems, USA). The RNA concentration and purity were measured using a NanoDrop™ 3100 Spectrophotometer (Thermo Scientific, USA). The RNA integrity was checked by agarose gel electrophoresis.

#### Semi-quantitative RT-PCR

First-strand cDNA was obtained after reverse transcription of 2 μg of total RNA with the superscript III first-strand synthesis cDNA Kit (Invitrogen, CA, USA) using the random primers supplied and following the manufacturer's instructions. The PCR was performed in a total reaction volume of 20 µl. Transcripts of the *etr1-1* gene were measured by semi-quantitative RT-PCR using 26S rRNA as the reference. The assays were performed with 0.3 μM of each primer and 2 μl of template cDNA. PCR conditions were same as genotyping, but had 36 cycles for *etr1-1* gene and 26 cycles for 26S rRNA. Standard dilutions of cDNA were used to assess the efficiency and quality of reactions. Negative controls (no template cDNA) were included in all semi-quantitative RT-PCR assays, and each assay was done in triplicate. PCR products were separated on 1.0% agarose gel and stained with safety gel stain (Applied Biosystems, USA).

#### Microarray hybridization

RNA from mixed samples of three biological replicates was used in microarray hybridization. At least 5 petals were harvested at random on each replicate. Double-stranded cDNA was synthesised using the Invitrogen SuperScript Double-Stranded cDNA Synthesis Kit (Invitrogen, USA). Briefly, double-stranded cDNA was synthesised using 10 μg of total RNA. The cDNA samples were labelled with one colour Cy3 random nonamers using the NimbleGen One-Colour DNA Labelling Kits (Roche, USA), followed by hybridisation to NimbleGen customized array slides (Roche, USA). Arrays were scanned by an MS 200 Microarray Scanner and data was collected and extracted by MS 200 Data collection and NimbleScan Software (Roche, USA).

### Data analysis

#### Differential gene analysis GO annotation

Differentially expressed genes were screened by 2-fold changes in comparison of samples with DEX versus without DEX at 24 h and 48 h. Gene sets were defined through biological processes primarily based on the following two database sources: TAIR (http://www.arabidopsis.org/) and Gene Ontology (http://www.geneontology.org/). According to the methods described by Dupuy [23], Fisher's exact test and the 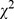
test were calculated to classify GO category. Enrichment analysis of gene sets, which determined whether a pre-defined set of genes show significantly up- or down-regulation compared with other gene sets [24], was performed to obtain more detailed and accurate function descriptions of differential genes by the formula: 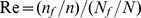, where 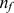 is the number of differential genes within the special category, 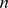 is the total number of genes within the same category, 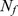
is the number of differential genes in the entire microarray, and 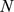
is the total number of genes in the microarray [25]. Genes classified to a significant GO category with the threshold of p-value <0.01 corrected by false discovery rate (FDR) were further analysed.

#### Time-serials cluster analysis

For time-course expression profiles, we firstly defined a set of independent models of expression profiles which are matched with practicable gene expression patterns over time according to RVM (Random variance model) corrective ANOVA [26], [27]. The ratio of signal density of individual time point to 0h was log normalised including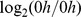, 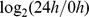, 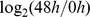. Each gene was ascribed to the model profile which represents the most possible expression profiles by correlation coefficient. The statistically significant profiles were calculated and then clustered by Fisher's act test and multiple comparisons.

#### Quantitative real-time PCR

One microgram of total RNA was reverse-transcribed using PrimeScript RT reagent with gDNA Eraser Kit (TaKaRA, Dalian), according to the manufacturer's instructions. In brief, one microgram of total RNA was digested with 2 μl 5×gDNA Eraser Buffer and 1 μl gDNA Eraser for 2 min at 42°C. The 10 μl reaction volume was reverse transcribed at 37°C for 15 min in a 20 μl reaction volume containing 4 μl 5×PrimeScript Buffer 2, 1 μl PrimeScript RT Enzyme Mix I and 1μl RT Primer Mix. The reaction was stopped by incubation at 85°C for 5s. One microliter of diluted 5× cDNA was used as template for PCR amplification using SYBR green Taq (TaKaRa, Dalian). Specific primers were designed by the primer 3 programme for the selected genes (listed in Table S3). Amplifications were performed in an Applied Biosystems 7300 system (Applied Biosystems, USA) with the following procedure: 2 min at 95°C, followed by 40 cycles of 45 s at 95°C, 30 s at 60°C, and 45 s at 72°C. Fluorescence was measured at the end of each cycle. Analysis of melting curves gave evidence of the absence of non-specific products and primer dimers. For data analysis, average threshold cycle (C_T_) values were calculated for each gene of interest, on the basis of three independent biological samples, as indicated [28], and were normalised and used to calculate relative transcript levels of the transcript as described elsewhere [29]. The 26S rRNA was used as an internal standard for normalisation [30].

### Statistical Analysis

Data were analysed by one-way ANOVA and SPSS 16.0 for windows. All experiments were replicated three times.

## Results

### Flower longevity and ethylene production in transgenic petunia plants

**Figure 1:**
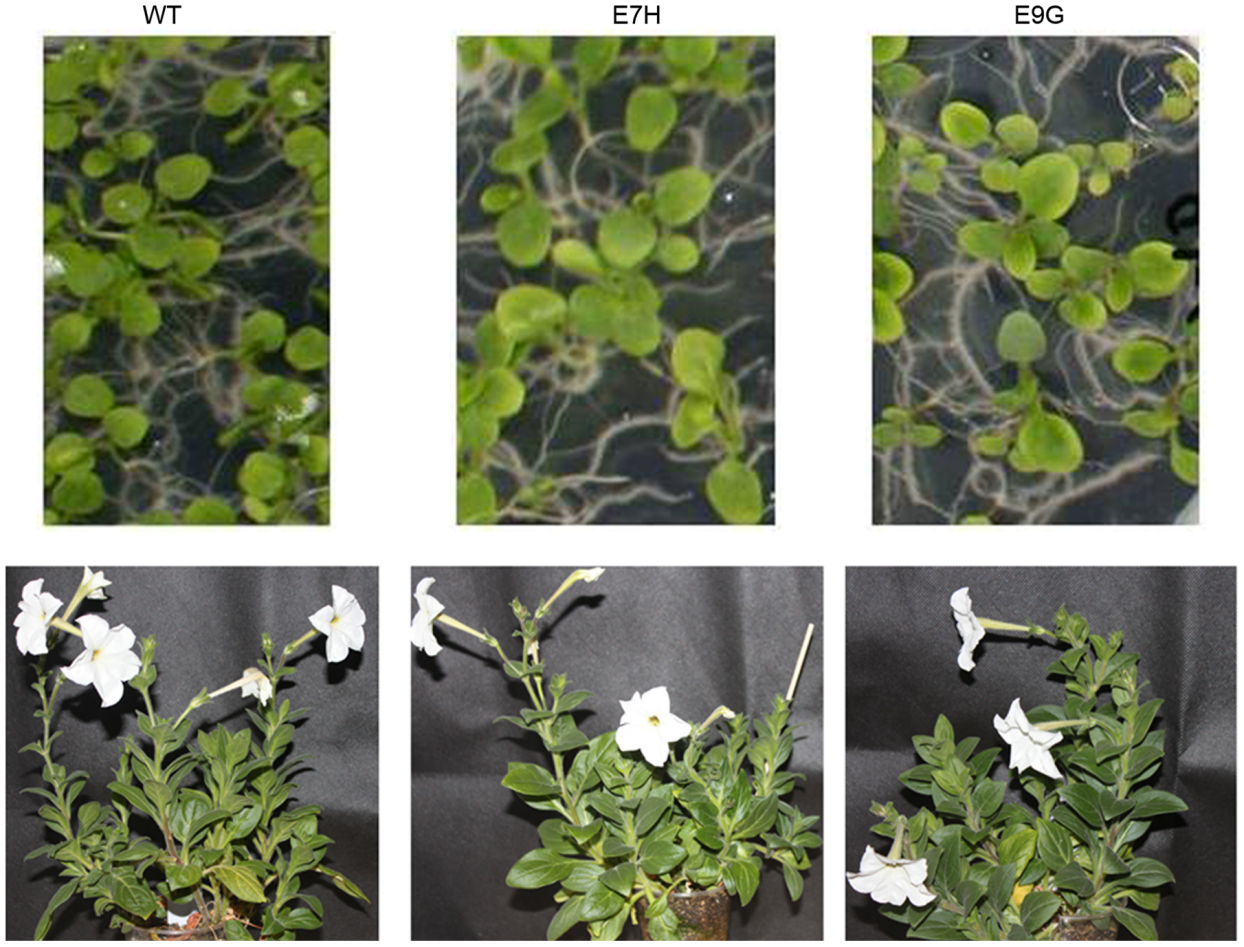
Representative seedlings and adult plants of transgenic petunia. A. Germination of T3 seeds of line E7H and line E9G on MS medium. B. Growth and development of line E7H and E9G under normal condition. WT was used as control.

The *Arabidopsis etr1-1* gene was cloned and successfully placed into a GVG inducible promoter construct. *Agrobacterium*-mediated transformation of petunia resulted in eleven transgenic lines, of which six lines of T3 homozygous transgenic plants were obtained. T3 transgenic plants displayed normal germination and the same growth and development phenotypes as wild type plants without DEX inducer (Fig. 1).

**Figure 2:**
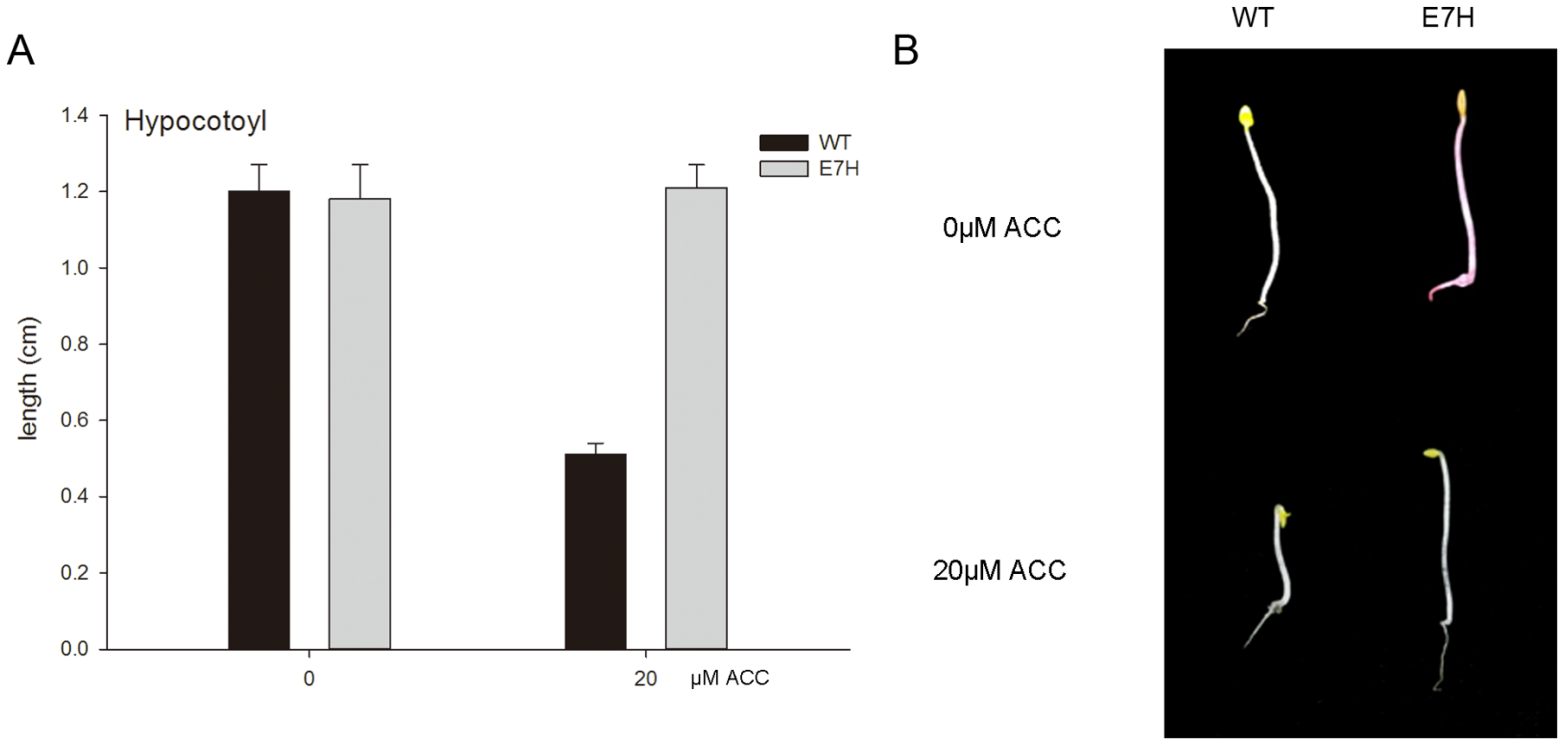
Induced *etr1-1* gene expression leads to transgenic plant insensitivity to ethylene. Seeds of WT and line E7H were planted on 30 μM DEX with or without 20 μM ACC and grown in the dark for 8 days. Quantitative measurements for hypocotyl lengths are shown in panel A. Values represent the means ±SD of at least 20 seedlings from three independent biological replicates. Representative seedlings of wild-type and transgenic line are displayed in panel B.

The triple response assay, which is characterised by distinguished morphological alterations including the radial expansion of the hypocotyls, the inhibition of hypocotyl elongation and the presence of an exaggerated apical hook [31], is known to select ethylene-related mutants of dark-grown seedlings. Wild-type and transgenic E7H seedlings grown on MS medium containing DEX and ACC in the dark were used to evaluate whether the induced *etr1-1* gene expression suppressed plant responses to ethylene. Transgenic seedlings treated with DEX showed a decreased sensitivity to ACC. The visible characteristics (apical hook, shortening and thickening of the hypocotyl) of the triple response were much more severe in the wild-type than in the DEX-treated transgenic seedlings (Fig. 2). The average hypocotyl length was 0.51 cm and 1.18 cm in wild-type and transgenic seedlings, respectively (Fig. 2). Hypocotyls of DEX-treated E7H seedlings were substantially longer than those of the wild-type seedlings. In the absence of ACC, E7H seedlings exhibited the same normal germination phenotype as wild-type seedlings (Fig. 2).

**Figure 3:**
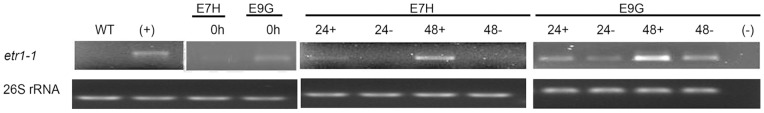
Semi-quantitative RT-PCR analysis of transcript levels of induced *etr1-1* gene expression. Petals of transgenic lines E7H and E9G were collected at 0h, 24 h and 48 h after with (+) or without DEX (−) treatments. WT petals and no template (−) were used as negative controls and plasmid DNA (+) was used as a positive control. 26S rRNA was used as an internal control.

We measured time-course expression profiles of the *etr1-1* gene on transgenic lines at 0 h, 24 h and 48 h at the absence or presence of the inducer by semi-quantitative RT-PCR. As shown in Fig. 3, the expression of *etr1-1* in the E7H line was not obviously detected at 0 h, although a low basal level of *etr1-1* transcription was observed in the E9G line under the same condition. Following prolonged induction time, the level of *etr1-1* gene expression was enhanced in either the E7H or the E9G line. Moreover, the transcriptional abundance of the *etr1-1* gene in the E9G line was apparently higher than that in the E7H line. This result illustrated that the GVG promoter activated by DEX leads to accumulations of time-dependent gradual increase of *etr1-1* transcription abundance.

**Figure 4:**
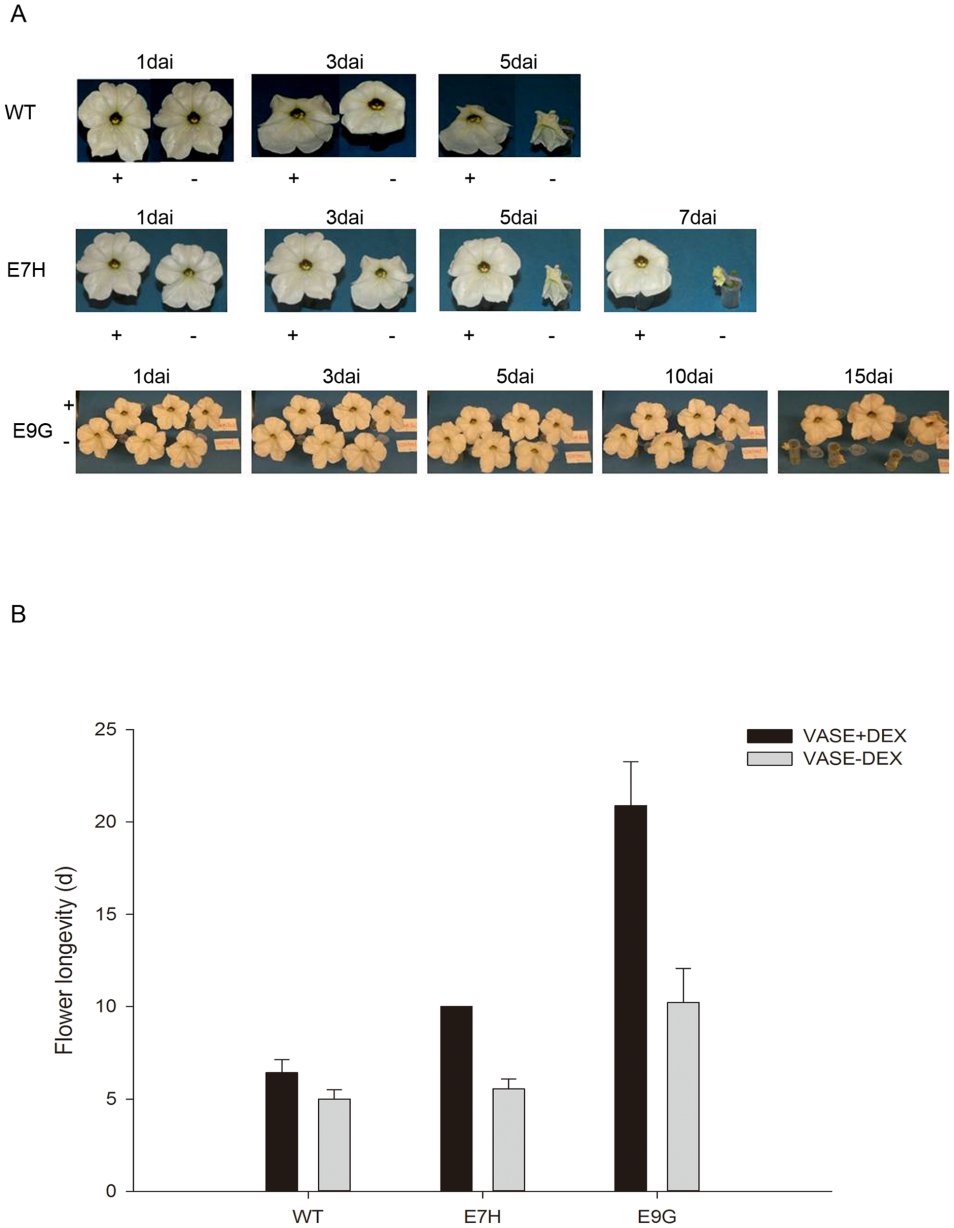
Inducible *etr1-1* gene expression extended flower longevity. Representative flowers either in the presence (+) or absence (−) of DEX were shown in the panel A. Flower longevities with the means ±SD are shown in the panel B. 20 flowers from the wild-type and each transgenic line were used for longevity evaluation.

To understand the effects of induced *etr1-1* expression on flower longevity, flowers that were fully opened but did not have dehisced anthers in E7H, E9G and wild-type lines were harvested in 2 ml tubes containing vase solution with or without 30 µM DEX. In the absence of inducer, visible senescence symptoms, such as wilting and curving of the petal edges, were observed after an average of 5–6 days in either the wild-type or transgenic flowers on E7H, whereas this occurred at 10 days on E9G. In the presence of the inducer, wild-type flowers normally exhibited wilting by 6–7 days on average, which was increased by 1–2 days depending on environmental conditions; however, flower longevity of E7H and E9G was extended by almost double, lasting average 12 days and 23 days, respectively (Fig. 4). Although basal gene expression and flower vase life are different between E7H and E9G lines, flower longevity of both E7H and E9G was extended by almost twice as many days as a function of DEX-induced the *etr1-1*expression.

**Figure 5:**
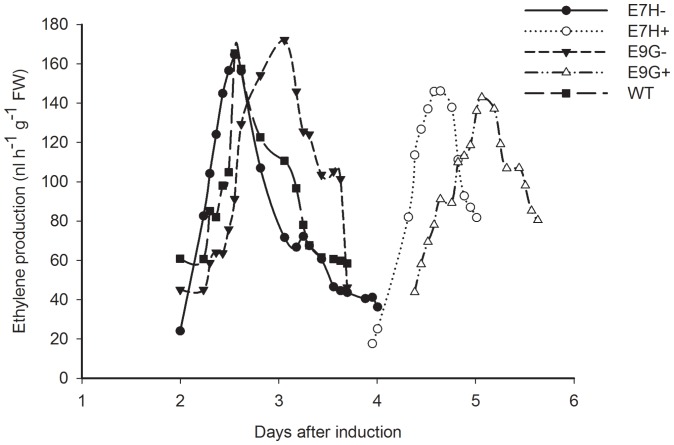
Delayed ethylene production by induced *etr1-1* gene expression. Ethylene productions from flowers of transgenic lines with (+) or without DEX (−) were monitored. WT was used as a control and empty cuvettes were used as a reference. Each independent experiment was repeated 3 times. Values represent the means of at least 6 flowers.

To illustrate whether ethylene production is regulated by the level of *etr1-1* gene expression, we measured ethylene production in detached E7H, E9G and wild-type flowers in the absence or presence of DEX by a real-time ethylene detection system EDT-300. In response to applied DEX, flowers from E7H and E9G showed a delayed peak value of ethylene production, which normally accompanies visual senescence symptoms (Fig. 5). The peak of ethylene production came at about 4 days and 5 days on flowers of E7H and E9G with DEX, respectively, compared with 2.5 days and 3 days on flowers of E7H and E9G without DEX, and 2.6 days for wide-type, respectively (Fig. 5). The results demonstrated that the gradually reduced and delayed ethylene production was controlled by the level of *etr1-1* gene expression.

### Differential gene expression on induced and non-induced transgenic flowers by DEX

In an attempt to elucidate the underlying mechanisms of extended flower longevity in transgenic petunia with induced expression of the *etr1-1* gene, we screened genes with differential expression at 24 h and 48 h with DEX-treatment versus without DEX-treatment. Gene ontology category enrichment analysis was utilised to identify possible biological processes. Significant functional categorisation was classified with *p*-value<0.01. The results showed that 5432 genes in the whole microarray were annotated to loci of *Arabidopsis* TAIR10 version by BLAST. At 24 h, 1902 genes were up-regulated and 1170 genes were down-regulated; and at 48 h, 818 genes were up-regulated and 2122 genes were down-regulated in comparison between E7H samples that were induced/non-induced by DEX (Table S1).

**Figure 6:**
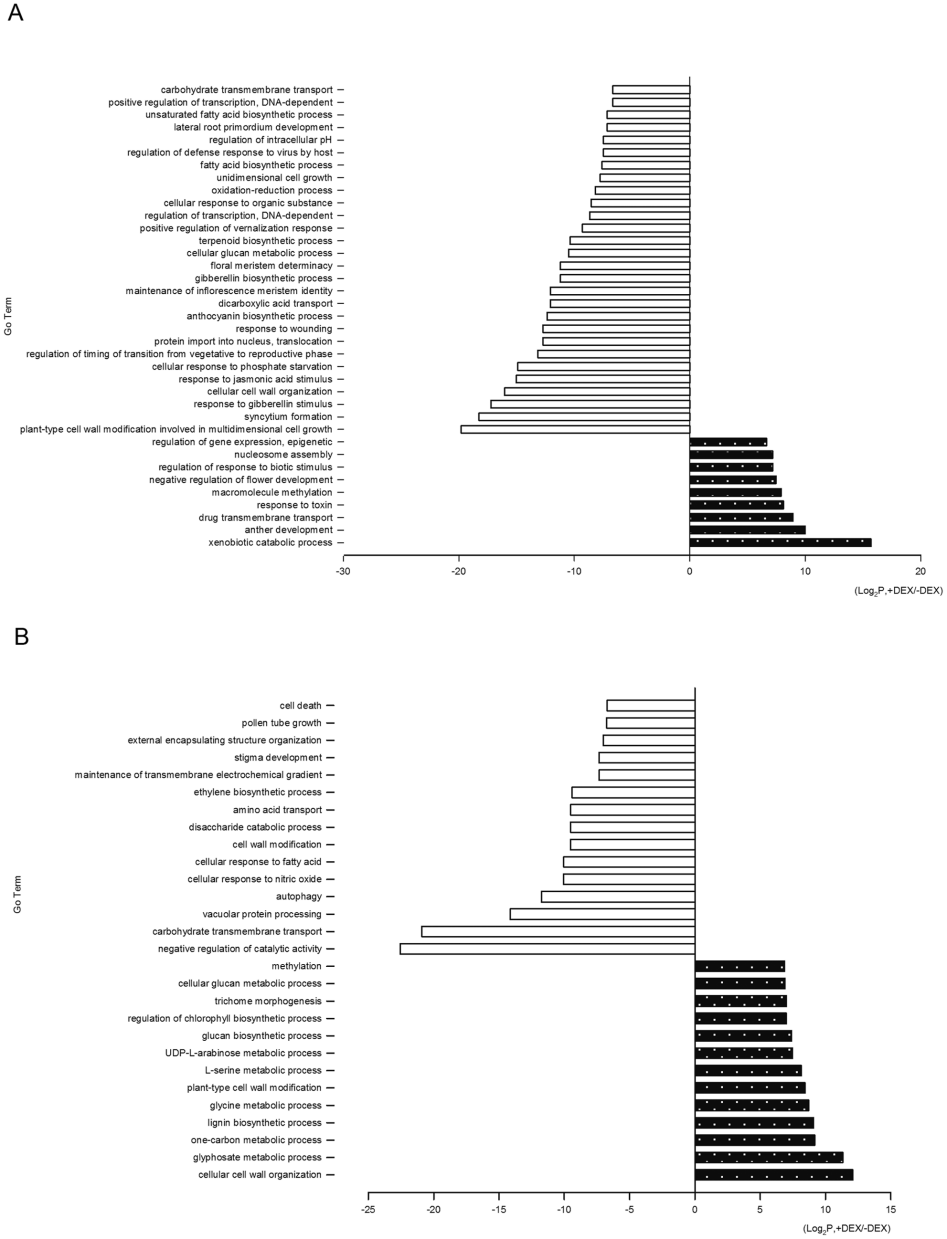
Distributions of GO term identified in comparisons of samples with and without DEX treatment. Gene ontology analysis of the differential genes in petals with (+) versus without (−) DEX at 24 h (A) and 48 h (B).

Differentially expressed gene sets at two time points were assigned to biological process categories on the basis of GO analysis. At 24 h, for the 147 annotated up-regulated genes, 34 fell into significant GO categories, including nine GO terms. For 413 annotated down-regulated genes, 360 were classified into 28 GO terms (Table S1 and Fig. 6A). Predominant up-regulated GO terms which included ‘anther development’, ‘negative regulation of flower development’ were listed in Table 1. The results illustrated that flowers stayed at flowering stage at 24 h after *etr1-1* induction expression. The GO categories for down-regulated genes contained ‘response of jasmonic acid and gibberellin stimulus’ and ‘gibberellin biosynthesis processes (Table 1). These results suggested induced *etr1-1* expression might repress the performance of JA and GA during ethylene-dependent flower senescence. Putative transcription factors, which accounted for 11.26% of the total genes of significant biological processes, mainly included MADS-box proteins, NAC domain proteins, homeodomain-like proteins (HD), B-box zinc finger proteins, bHLH DNA-binding protein, MYB domain proteins, and were down-regulated. A significantly high proportion of ‘cell wall modification’ was observed in down-regulated GO categories (Table 1).

**Table 1:** Summarized results from GO category based on biological process for differentially expressed genes in comparisons of samples with DEX (+)/without DEX (−) at 24 h.

Putative function	Number		Type of expression
GO:0006355 regulation of transcription, DNA-dependent			
AUX/IAA transcriptional regulator family protein	1		DOWN
Auxin response factor 19	2		DOWN
basic helix-loop-helix (bHLH) DNA-binding family protein	4		DOWN
B-box type zinc finger family protein	4		DOWN
BIG PETAL P	2		DOWN
CONSTANS-like	2		DOWN
Cycling DOF factor	1		DOWN
DNA glycosylase superfamily protein	1		DOWN
ERF domain protein 9	1		DOWN
Floral meristem identity control protein LEAFY (LFY)	1		DOWN
Homeodomain-like superfamily protein	5		DOWN
Indole-3-acetic acid 7	2		DOWN
Integrase-type DNA-binding superfamily protein	2		DOWN
Jasmonate-zim-domain protein 1	1		DOWN
K-box region and MADS-box transcription factor family protein	7		DOWN
MYB domain protein	4		DOWN
NAC domain transcriptional regulator superfamily protein	6		DOWN
Phytochrome interacting factor 4	1		
Tubby like protein 2	1		DOWN
WRKY DNA-binding protein	3		DOWN
GO:0009826 cell wall modification			
Expansin	12		DOWN
Xyloglucan endotransglycosylase	8		DOWN
Galacturonosyltransferase-like	2		DOWN
Glycosyl hydrolase superfamily protein	5		DOWN
COBRA-like extracellular glycosyl-phosphatidyl Inositol-anchored protein family	11		DOWNDOWN
Leucine-rich repeat (LRR) family protein	1		DOWN
GO:0009739 response to gibberellin stimulus			
Xyloglucan endotransglycosylase 6		2	DOWN
Floral meristem identity control protein LEAFY (LFY)		1	DOWN
Expansin A4		3	DOWN
Myb domain protein 3		1	DOWN
Homeodomain-like superfamily protein		3	DOWN
K-box region and MADS-box transcription factor family protein		4	DOWN
GO:0009686 gibberellin biosynthetic process			
Ent-kaurenoic acid hydroxylase 2		3	DOWN
GO:0009753 response to jasmonic acid stimulus			
Myb domain protein 113		3	DOWN
Major facilitator superfamily protein		3	DOWN
Indole-3-acetic acid 7		1	DOWN
Homeodomain-like superfamily protein		4	DOWN
Jasmonate-zim-domain protein 1		1	DOWN
Glutamate-cysteine ligase		1	DOWN
Leucoanthocyanidin dioxygenase		1	DOWN
GO:0034219 carbohydrate transmembrane transport			
Glucose-6-phosphate/phosphate translocator-related		2	DOWN
Major facilitator superfamily protein		1	DOWN
Senescence-associated gene 29		3	DOWN
Polyol/monosaccharide transporter 5		1	DOWN
GO:0048653 anther development			
Cytochrome P450, family 94, subfamily B, polypeptide 1		1	UP
C2H2 and C2HC zinc fingers superfamily protein		2	UP
GO:0009910 negative regulation of flower development			
Zinc finger (C2H2 type) family protein		2	UP

A more elaborate version of this table is available as supplementary data (Table S1).

At 48 h, among the 108 annotated up-regulated genes, 76 genes were classified into 15 GO terms, and among the 429 annotated down-regulated genes, 119 genes fell into significant GO categories (Table S1 and Fig. 6B). The GO categories for up-regulated genes mainly contained lignin biosynthesis process and cell wall modification which was composed of ‘cell wall biological processes’ such as ‘cell wall organisation’, ‘one-carbon metabolic processes’, and ‘glyphosate metabolic processes’ (Table 2 and Table S1); for down-regulated genes they consisted of ‘carbohydrate transmembrane transport’, ‘vacuolar protein processing’, ‘autophagy’, ‘ethylene biosynthesis processes’, ‘cell wall modification’ and ‘cell death’ (Table 2). The differentially expressed genes involved in significant GO categories are summarised in Table S1.

**Table 2:** Summarized results from GO category based on biological process for differentially expressed genes in comparisons of samples with DEX (+)/without DEX (−) at 48 h.

Putative function	Number	Type of expression
GO:0009693 ethylene biosynthetic process		
ethylene-forming enzyme	8	DOWN
1-amino-cyclopropane-1-carboxylate synthase 2	1	DOWN
GO:0034219 carbohydrate transmembrane transport		
Major facilitator superfamily protein	6	DOWN
inositol transporter 4	1	DOWN
senescence-associated gene 29	4	DOWN
Nodulin MtN3 family protein	6	DOWN
GO:0006624 vacuolar protein processing		
gamma vacuolar processing enzyme	5	DOWN
GO:0006914 autophagy		
Ubiquitin-like superfamily protein	6	DOWN
GO:0045229 external encapsulating structure organization		
Plant invertase/pectin methylesterase inhibitor superfamily	8	DOWN
Leucine-rich repeat (LRR) family protein	1	DOWN
xyloglucan endotransglucosylase/hydrolase 25	1	DOWN
Pectin lyase-like superfamily protein	10	DOWN
GO:0008219 cell death		
alcohol dehydrogenase 1	2	DOWN
ADR1-like 1	1	DOWN
elicitor-activated gene 3-2	1	DOWN
Seven transmembrane MLO family protein	2	DOWN
lipid phosphate phosphatase 3	1	DOWN
cinnamyl alcohol dehydrogenase 9	2	DOWN
Kunitz family trypsin and protease inhibitor protein	1	DOWN
fatty acid hydroxylase 1	1	DOWN
GO:0009809 lignin biosynthetic process		
S-adenosyl-L-methionine-dependent methyltransferases superfamily protein	5	UP
GO:0009827 plant-type cell wall modification		
expansin B3	3	UP
xyloglucan endotransglucosylase/hydrolase 16	3	UP
reversibly glycosylated polypeptide 1	2	UP
debranching enzyme 1	1	UP

A more elaborate version of this table is available as supplementary data (Table S1).

### Time-course expression profiles analysis in transgenic plants with or without DEX treatment

To further narrow the target genes which were changed due to induced *etr1-1* gene expression, we focused on comparing time-course expression trends between the two treatments, where the *log_2_* ratio of gene expression for every time point versus 0h was corresponded to the following x-axis order: 0 h, 24 h and 48 h.

**Figure 7:**
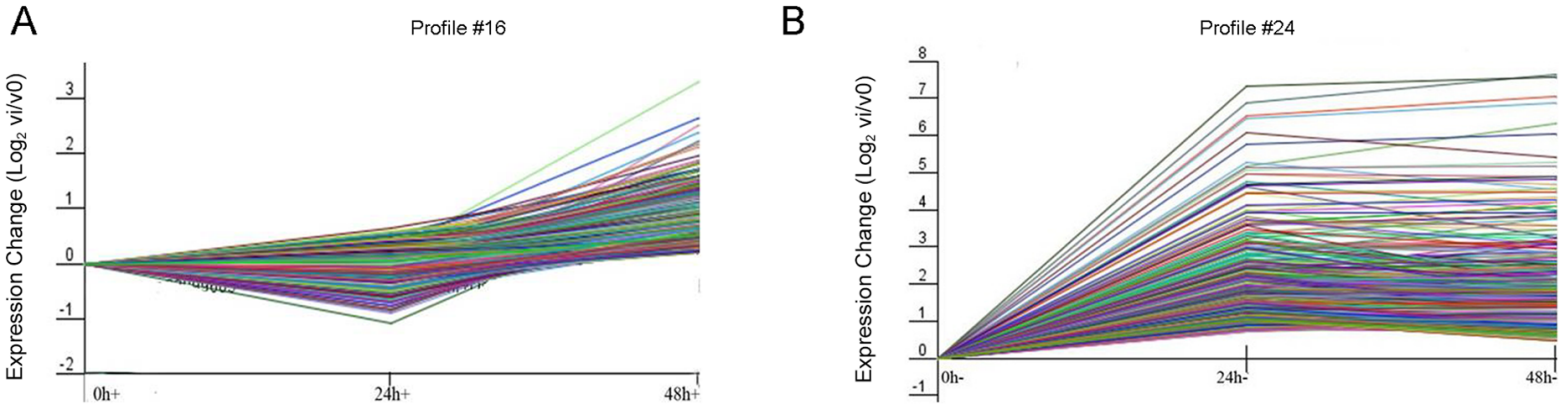
Expression profiles of genes from samples with (+) or without (−) DEX treatment. The horizontal axis represented given time points with (+, left panel) or without DEX (-, right panel) treatment, and the vertical axis shows the time series of expression levels for the gene after Log normalised transformation.

Seven model profiles were obtained to summarise the expression patterns. Two totally opponent trends of time-course gene expression between petals with and without induced *etr1-1* expression were selected for GO analysis. As shown in Fig. 7, a stable-then-up pattern for petals with DEX-treatment for profile No.16, and an up-then-stable pattern for petals without DEX-treatment were observed for profile No.24 (Fig. 7). In these two profiles, GO categories were predominant in ‘the regulation of transcription’, which included genes encoding MYB-like DNA binding protein, a zinc finger transcription factor, homeobox-leucine zipper family protein, C2H2-like zinc finger protein, MADS-box transcription factor family protein, NAC domain containing protein, GATA transcription factor, and WUS-interacting protein (Table S2).

### Quantitative real-time PCR analysis

**Figure 8:**
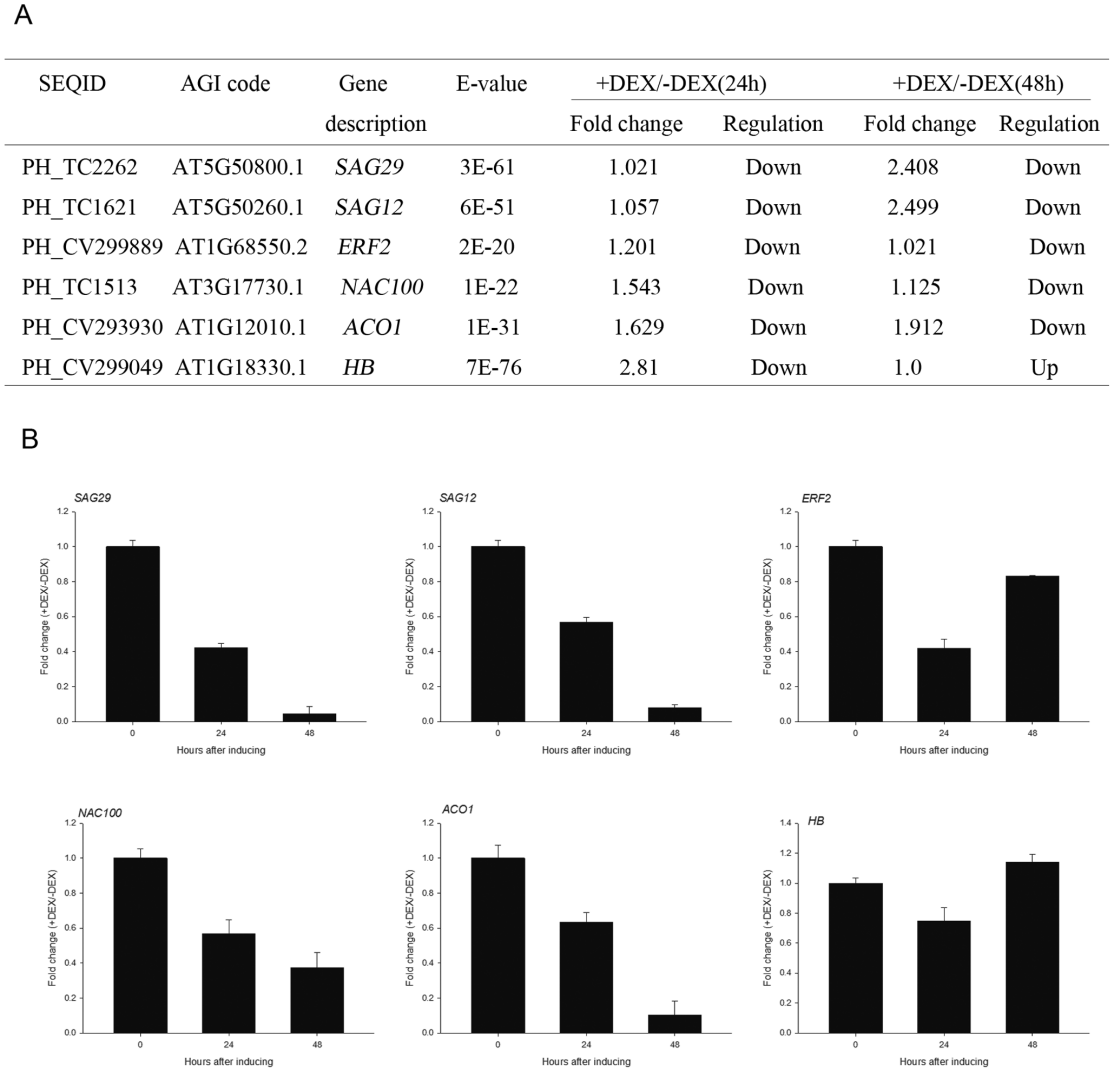
Validation of microarray data by quantitative real-time PCR. Six genes were selected and their time-course expression profiles were evaluated by quantitative real-time PCR in samples with (+) or without (−) DEX at given time points. (A) Fold changes were obtained from microarray analysis. (B) Fold changes were obtained from qRT-PCR analysis. Relative expression was obtained using 26S rRNA as an internal control. cDNAs were synthesised from three biological replicates.

To confirm our microarray data, we selected six genes to test expression by quantitative real-time PCR. As shown in Fig. 8, for the selected genes, results of quantitative real-time PCR matched the microarray data very well, thereby validating the microarray analysis. The expression of 1-aminocyclopropane-1-carboxylic acid oxidase (ACO) converts ACC to ethylene [32], which is a critical enzyme in the ethylene biosynthesis pathway, was repressed in petals with DEX treatment. Expression of ethylene response factor 9 (*ERF9*), a downstream component of the ethylene signalling pathway [33], was substantially suppressed in petals with DEX-treatment than in those without DEX-treatment at 24 h and 48 h. *HD,* which encodes a homeodomain-like superfamily protein, exhibited significantly decreased expression levels in petals with DEX treatment at 24 h compared to those without DEX treatment. The expression levels of *SAG29* and *SAG12*, which are senescence-associated genes [34], as well as *NAC100*, were repressed and obtained the lowest after 48h in petals with DEX treatment.

## Discussion

### Controlling *etr1-1* gene expression and flower senescence by chemical inducible system

Our first goal was to investigate floral senescence by an inducible system. Previous research had established the effectiveness of heterologous expression of the mutant *etr1-1* receptor in Petunia [7]. However, 35S:*etr1-1* petunia displayed defective growth and development. In addition, plants, which had been transformed *etr1-1* with its own promoter, showed abnormal development and altered pathogen sensitivity. For example, transgenic soybeans with *etr1-1* have been demonstrated increased or decreased resistant to pathogen determined by different strains [35]. Ethylene-insensitive tobacco plants were much more susceptible to pathogen than wild-type plants [15].

We wished to avoid the negative side-effects observed when expressing this gene constitutively while still avoiding the limitations of the tissue specific promoter. This research has demonstrated that an inducible system can effectively be used to regulate expression of the mutant receptor *etr1-1,* and thereby control floral senescence in ethylene-dependent senescing flowers. E7H and E9G lines, which exhibited ethylene insensitivity only when the inducer was applied, were selected to avoid the undesirable effects of ethylene insensitivity on germination and other key events. The vase life of flowers was extended by an average of over 6–10 days, depending on the level of *etr1-1* gene expression.

The vase life of E9G flowers was considerably longer than that of E7H flowers either with or without DEX. The results further confirmed that flower longevity awarded by the *etr1-1* gene is related to the level of its expression. Although transgenic E9G line gave an evidence of low gene expression without inducer, its ethylene sensitivity was tested in our initial “leaky” experiments (data not shown). Gallie [36] confirmed a linear relationship between the degree of insensitivity and *etr1-1* expression. Though that remains to be elucidated, we conclude that gene expression of E9G line is too low to have ethylene insensitivity symptom while without inducer.

### Time-course gene expression changes associated with induced *etr1-1* expression

An extra benefit of using an inducible system is that gene expression of interest can be much tightly, temporally and spatially controlled. The lines, which were generated for this study, allow expression of *etr1-1* in an exact specific manner, and thus can be of use in investigating underlying molecular mechanisms and the timing of ethylene dependent events during floral senescence.

At 24h after *etr1-1* induced expression, transcripts of 50 genes encoded proteins involved in the regulation of transcription including 20 putative transcription factors were down-regulated. Of those transcription factors, homeodomain-like superfamily protein (LHY), B-box zinc finger family protein and bHLH exhibited up-regulated expression during nature flower senescence in petunia. Similar types of transcription factors were also up-regulated during flower senescence of *Dianthus caryophyllus* [37]. We also found down-regulated expression of Integrase-type DNA-binding superfamily protein, BIG PETAL P, CONSTANS-like protein, Tubby like protein, Cycling DOF factor and Phytochrome interacting factor which have not been reported during flower senescence so far. The possible function of these genes in the ethylene-regulation of senescence is not yet clear.

Other down-regulated putative transcription factors shared high homology with NAC domain-containing proteins 2 and 100, WRKY40, AGL20 and AGL24 of *Arabidopsis*. NAC transcription factors comprise a superfamily containing 109 members, of which *AtNAP* and *NAC2* were known to be involved in leaf senescence in *Arabidopsis* [38]–[40]. WRKY transcription factors comprise a large gene family, which controls senescence processes, and include WRKY6 [41], WRKY4, WRKY70 [42], WRKY53 [43] and WRKY22 [44]. Transcriptome analysis of leaf senescence in *Arabidopsis* suggested that transcription factors including NAC and WRKY played an important role in regulating differential gene expression [45]. MADS-box domain proteins, such as AGL15, when over-expressed around the time of flower opening resulted in delayed senescence of floral organs [46], [47]. The expression of all known MADS-box genes in the *Arabidopsis* genome was not monitored in senescing leaves [45]. Other transcription factors including AP2, MYB, HB, bZIP, bHLH were observed in senescing leaves of *Arabidopsis*. However, HMG-box, CCAAT, HSF, SBP, TUB, JUMONJI, Alfin-like and PcG was found in senescing leaves of *Arabidopsis* [45] but not in senescing flowers of petunia. These results demonstrated that petal senescence might share the similar genetic mechanism as that of leaf senescence to some extent. Nevertheless, that the regulation of different transcription factors was observed between flowers and leaves might be due to different organs.

Other putative transcription factors which shared high similarity with MYB-DNA binding protein (MYB3, MYB6, MYB113, MYB70) [48], indole-3acetic acid (IAA7), Aux/IAA (SHY2) [48], [49], B-box type zinc finger family protein (AT4G2723), and ARF19 [50], which was involved in the ethylene pathway and regulated by ethylene treatment [51], were down-regulated at 24 h. Notably, a putative transcription factor defined as ERF9, which is a downstream gene of the *EIN/EIL* transcription factor in the ethylene pathway, was down-regulated at 24 h by induction of *etr1-1* expression. This is consistent with another report suggested that a gene which shared high homology to *ERF2* displayed up-regulated expression during daffodil petal senescence and *Arabidopsis* leaf senescence [52], [53].

Of these transcription factors, NAC, MYB, MADS-box, Aux/IAA, were up-regulated at the first day during carnation petal senescence [48]. However, C2H2 zinc finger superfamily protein (SUF4), which was up-regulated expression of C2H2 in *Alstroemeria pelegrina* [54] and *Mirabilis jalapa* petal senescence [55], displayed up-regulated expression after 48h with induced *etr1-1* expression. This suggested that transcript activation of putative transcription factors was delayed by induced *etr1-1* expression. Moreover, time-series transcription analysis further demonstrated that the reason why transcription factors were down-regulated during the initial 24 h in the case of induced *etr1-1* expression was due to up-regulated expression of these genes when there was a lack of induced *etr1-1* expression.

Treatment with GA_3_ extended the longevity of cut carnation [56] and daffodil flowers [57]. The effect of the application of JA on the flower longevity was dependent on species; for example it hastened flower senescence in petunia hybrids, *Dendrobium*, and *Phalaenopsis* [58], [59] but did not change the flower longevity of *Petunia inflate* [60]. In our experiments, GO analysis suggested that induced *etr1-1* expression affected the performance of the GA and JA pathways after 24h in ethylene-dependent petunia flower senescence. These results also provided a hint that hormone crosstalk was involved in regulating the senescence of ethylene-sensitive flowers.

ACC synthase and ACC oxidase are regarded as crucial limiting enzymes for ethylene biosynthesis [61]. During petal senescence in *Dinathus caryphyllus*, genes encoding ACS and ACO are up-regulated [62]. Transcript analysis suggested a suppressed effect of induced *etr1-1* expression on the expression pattern of putative ACS2, ACO1 and ACO2 after 48h. Transcription analysis of *Arabidopsis* illustrated that genes encoding ACC synthases and ACC oxidase were up-regulated during leaf senescence [53], suggesting that even on ethylene-sensitive plants species ethylene might not trigger but hasten the senescence process of flower as well as leaf [63]. The results also provided a reasonable explanation for the delayed peaks of ethylene production in flowers with induced *etr1-1* expression.

Cell wall modification was known to be a part of petal senescence [1]. Expansins and xyloglucan endotransglucosylase synergistically performed cell wall modifications [64]. Transcriptional abundance of endoxyloglucan transferase in *Alstoemeria pelegrinwas* [54], expansin in *Dianthus caryophyllus* [48], and xyloglucan endo-transglycolase/hydrolose (XET/XTH/XTR) in *Iris×hollandica* [65] were increased during flower senescence. Cell wall metabolism, including ‘cell wall modification’, ‘multidimensional cell growth’, ‘plant-type cell wall organisation’, ‘cell wall loosening’, ‘cell wall organisation’, and ‘cellular glucan metabolic processes’ were suppressed due to induced *etr1-1* expression after 24h and then up-regulated after 48h. The major genes involved in these GO categories consisted of putative *EXP11*, *EXPA4*, *EXPA8*, *XTR7* and *MERI5B*. A putative gene encoding a plant invertase/pectin methylesterase inhibitor superfamily protein was down-regulated at 48h. These results indicated that induced *etr1-1* expression suppressed and retarded transcriptional accumulation of these genes at late stage during ethylene-dependent petal senescence.

Senescence-related biological processes, including ‘carbohydrate transmembrane transport’, ‘vacuolar protein processing’, ‘autophagy’, and ‘cell death’, were down-regulated by induced *etr1-1* expression during the monitored period. High level of transcriptional abundance of *MtN3* was found in the late stages of Iris flower senescence [65]. Either putative *MtN3* or *SAG29* genes encoding senescence-associated proteins that have a structural organisation similar to those of MtN3 proteins were down-regulated after induced *etr1-1* expression at the late stage. *Arabidopsis* plants with over-expression of *SAG2*9 gene displayed accelerated senescence [34]. Vacuolar processing enzyme (VPE), which is known to be a caspase-like protein [48], [66] and exhibits homology to cysteine protease, a senescence-associated protein, was repressed after 48 h at the presence of DEX. Research illustrated that autophagy displayed a sudden and rapid increase during leaf senescence [1]. The putative *APG8A* gene encoding an ubiquitin-like protein which participates in the process of autophagy was down-regulated by induced *etr1-1* expression after 48 h.

In conclusion, because ethylene plays important roles in multi-aspects of plant growth and development and the mutation of ethylene receptor under constitutive expression interferes with many physiological processes, the ability to switch on or off ethylene insensitivity on specific tissues and at a particular time point by adding or removing the inducer offers more effective ways to study ethylene-dependent flower senescence. Down-regulated expression of putative genes encoding ‘regulation of transcription’ at early stage of induced *etr1-1* expression gave us a hint that transcription factors or regulators might play important roles in the process of extended flower longevity. This provides us a research direction for the future to investigate functions of putative transcription factors such as NAC, HD, bHLH and MYB on ethylene-dependent flower senescence. In addition, down-regulated expression of gene encoding proteins in the cell wall modification and the process of senescence were consistent with visible delayed senescence phenotype of flowers with induced *etr1-1* expression.

## Supporting Information

Table S1 GO annotation for differentially expressed genes between samples with DEX and without DEX at given time point.
(XLSX)

Table S2 GO annotaion for time-course expression profile #16 and #24.
(XLSX)

Table S3 Gene-specific primers for quantitative real-time PCR.
(DOCX)

## References

[1] van Doorn WG, Woltering EJ (2008) Physiology and molecular biology of petal senescence. J Exp Bot 59: 453–480. 18310084 10.1093/jxb/erm356

[2] Gerats T, Vandenbussche M (2005) A model system for comparative research: Petunia. Trends Plant Sci 10: 251–256. 15882658 10.1016/j.tplants.2005.03.005

[3] Bleecker AB, Estelle MA, Somerville C, Kende H (1988) Insensitivity to ethylene conferred by a dominant mutation in *Arabidopsis thaliana* . Science 241: 1086–1089. 17747490 10.1126/science.241.4869.1086

[4] Bleecker AB, Kende H (2000) Ethylene: a gaseous signal molecule in plants. Annu Rev Cell Dev Biol 16: 13–18. 10.1146/annurev.cellbio.16.1.1 11031228

[5] Chang C, Kwok S, Bleecker A, Meyerowitz E (1993) Arabidopsis ethylene-response gene ETR1: similarity of product to two-component regulators. Science 262: 539–544. 8211181 10.1126/science.8211181

[6] Schaller GE, Bleecker AB (1995) Ethylene-binding sites generated in yeast expressing the Arabidopsis ETR1 gene. Science 270: 1809–1811. 8525372 10.1126/science.270.5243.1809

[7] Wilkinson JQ, Lanahan MB, Clark DG, Bleecker AB, Chang C, et al (1997) A dominant mutant receptor from Arabidopsis confers ethylene insensitivity in heterologous plants. Nat Biotech 15: 444–447. 10.1038/nbt0597-444 9131623

[8] Langston BJ, Bai S, Jones ML (2005) Increases in DNA fragmentation and induction of a senescence-specific nuclease are delayed during corolla senescence in ethylene-insensitive (*etr1-1*) transgenic petunias. J Exp Bot 56: 15–23. 15475372 10.1093/jxb/eri002

[9] Sanikhani M, Mibus H, Stummann BM, Serek M (2008) Kalanchoe blossfeldiana plants expressing the Arabidopsis etr1-1 allele show reduced ethylene sensitivity. Plant Cell Rep 27: 729–737. 18080125 10.1007/s00299-007-0493-6

[10] Sriskandarajah S, Mibus H, Serek M (2007) Transgenic *Campanula carpatica* plants with reduced ethylene sensitivity. Plant Cell Rep 26: 805–813. 17221226 10.1007/s00299-006-0291-6

[11] Clark DG, Gubrium EK, Barrett JE, Nell TA, Klee HJ (1999) Root formation in ethylene-insensitive plants. Plant Physiol 121: 53–60. 10482660 10.1104/pp.121.1.53 PMC59389

[12] Clevenger DJ, Barrett JE, Klee HJ, Clark DG (2004) Factors affecting seed production in transgenic ethylene-insensitive petunias. J Am Soc Hortic Sci 129: 401–406.

[13] Gubrium EK, Clevenger DJ, Clark DG, Barrett JE, Nell TA (2000) Reproduction and Horticultural Performance of Transgenic Ethylene-insensitive Petunias. J Am Soc Hortic Sci 125: 277–281.

[14] Serek M, Woltering E, Sisler E, Frello S, Sriskandarajah S (2006) Controlling ethylene responses in flowers at the receptor level. Biotechnol Adv 24: 368–381. 16584864 10.1016/j.biotechadv.2006.01.007

[15] Knoester M, Van Loon LC, Van den Heuvel J, Hennig J, Bol JF, et al (1998) Ethylene-insensitive tobacco lacks non-host resistance against soil-borne fungi. P Natl Acad Sci USA 95: 1933–1937. 10.1073/pnas.95.4.1933 PMC19216 9465120

[16] Moore I, Samalova M, Kurup S (2006) Transactivated and chemically inducible gene expression in plants. Plant J 45: 651–683. 16441354 10.1111/j.1365-313X.2006.02660.x

[17] Aoyama T, Chua NH (1997) A glucocorticoid-mediated transcriptional induction system in transgenic plants. Plant J 11: 605–612. 9107046 10.1046/j.1365-313x.1997.11030605.x

[18] Mahmood T, Zar T, Naqvi SMS (2008) Multiple pulses improve electroporation efficiency in Agrobacterium tumefaciens. Electron J Biotech, North America, 1115.

[19] Murray MG, Thompson WF (1980) Rapid isolation of high molecular weight plant DNA. Nucleic Acids Res 8: 4321–4325. 7433111 10.1093/nar/8.19.4321 PMC324241

[20] Craft J, Samalova M, Baroux C, Townley H, Martinez A, et al. (2005) New pOp/LhG4 vectors for stringent glucocorticoid-dependent transgene expression in *Arabidopsis*. Plant J. 41, 899–918. 10.1111/j.1365-313X.2005.02342.x 15743453

[21] Benlloch-González M, Romera J, Cristescu S, Harren F, Fournier JM, et al (2010) K^+^ starvation inhibits water-stress-induced stomatal closure via ethylene synthesis in sunflower plants. J Exp Bot 61: 1139–1145. 20054030 10.1093/jxb/erp379

[22] Salman A, Filgueiras H, Cristescu S, Lopez-Lauri F, Harren F, et al. (2009) Inhibition of wound-induced ethylene does not prevent red discoloration in fresh-cut endive *Cichorium intybus* L. Eur Food Res Technol 228, 651–657.

[23] Dupuy D, Bertin N, Hidalgo CA, Venkatesan K, Tu D, et al (2007) Genome-scale analysis of in vivo spatiotemporal promoter activity in Caenorhabditis elegans. Nat Biotechnol 25: 663–668. 17486083 10.1038/nbt1305

[24] Ge H, Wei M, Fabrizio P, Hu J, Cheng C, et al (2010) Comparative analyses of time-course gene expression profiles of the long-lived sch9Delta mutant. Nucleic Acids Res 38: 143–158. 19880387 10.1093/nar/gkp849 PMC2800218

[25] Schlitt T, Palin K, Rung J, Dietmann S, Lappe M, et al (2003) From gene networks to gene function. Genome Res 13: 2568–2576. 14656964 10.1101/gr.1111403 PMC403798

[26] Ramoni MF, Sebastiani P, Kohane IS (2002) Cluster analysis of gene expression dynamics. P Natl Acad Sci USA 99: 9121–9126. 10.1073/pnas.132656399 PMC123104 12082179

[27] Miller LD, Long PM, Wong L, Mukherjee S, McShane LM, et al (2002) Optimal gene expression analysis by microarrays. Cancer Cell 2: 353–361. 12450790 10.1016/s1535-6108(02)00181-2

[28] Balaji V, Mayrose M, Sherf O, Jacob-Hirsch J, Eichenlaub R, et al (2008) Tomato transcriptional changes in response to *Clavibacter michiganensis* subsp. *michiganensis* reveal a role for ethylene in disease development. Plant Physiol 146: 1797–1809. 18245454 10.1104/pp.107.115188 PMC2287351

[29] Pfaffl MW (2001) A new mathematical model for relative quantification in real-time RT-PCR. Nucleic Acids Res 29: e45. 11328886 10.1093/nar/29.9.e45 PMC55695

[30] Wang H, Liu G, Li C, Powell ALT, Reid MS, et al. (2013) Defence responses regulated by jasmonate and delayed senescence caused by ethylene receptor mutation contribute to the tolerance of petunia to *Botrytis cinerea*. Mol Plant Pathol Published online Feb. 10.1111/mpp.12017 PMC6638649 23437935

[31] Gallie DR (2010) Regulated ethylene insensitivity through the inducible expression of the Arabidopsis *etr1-1* mutant ethylene receptor in tomato. Plant Physiol 152: 1928–1939. 20181754 10.1104/pp.109.151688 PMC2850004

[32] Kende H (1993) Ethylene biosynthesis. Ann Rev Plant Physiol Plant Mol Biol 44: 283–307.

[33] Lin Z, Zhong S, Grierson D (2009) Recent advances in ethylene research. J Exp Bot 60: 3311–3336. 19567479 10.1093/jxb/erp204

[34] Seo PJ, Park JM, Kang SK, Kim SG, Park CM (2011) An Arabidopsis senescence-associated protein SAG29 regulates cell viability under high salinity. Planta 233: 189–200. 20963606 10.1007/s00425-010-1293-8

[35] Hoffman T, Schmidt JS, Zheng X, Bent AF (1999) Isolation of ethylene-insensitive soybean mutants that are altered in pathogen susceptibility and gene-for-gene disease resistance. Plant Physiol 119: 935–950. 10069832 10.1104/pp.119.3.935 PMC32108

[36] Gallie DR (2010) Regulated ethylene insensitivity through the inducible expression of the Arabidopsis *etr1-1* mutant ethylene receptor in tomato. Plant Physiol 152: 1928–1939. 20181754 10.1104/pp.109.151688 PMC2850004

[37] Hoeberichts FA, van Doorn WG, Vorst O, Hall RD, van Wordragen MF (2007) Sucrose prevents up-regulation of senescence-associated genes in carnation petals. J Exp Bot 58: 2873–2885. 17630294 10.1093/jxb/erm076

[38] Guo Y, Gan S (2006) AtNAP, a NAC family transcription factor, has an important role in leaf senescence. Plant J 46: 601–612. 16640597 10.1111/j.1365-313X.2006.02723.x

[39] Balazadeh S, Siddiqui H, Allu AD, Matallana-Ramirez LP, Caldana C, et al (2010) A gene regulatory network controlled by the NAC transcription factor ANAC092/AtNAC2/ORE1 during salt-promoted senescence. Plant J 62: 250–264. 20113437 10.1111/j.1365-313X.2010.04151.x

[40] Kim JH, Woo HR, Kim J, Lim PO, Lee IC, et al (2009) Trifurcate feed-forward regulation of age-dependent cell death involving miR164 in Arabidopsis. Science's STKE 323: 1053. 10.1126/science.1166386 19229035

[41] Robatzek S, Somssich IE (2002) Targets of AtWRKY6 regulation during plant senescence and pathogen defense. Gene Dev 16: 1139. 12000796 10.1101/gad.222702 PMC186251

[42] Ülker B, Shahid Mukhtar M, Somssich I (2007) The WRKY70 transcription factor of Arabidopsis influences both the plant senescence and defense signaling pathways. Planta 226: 125–137. 17310369 10.1007/s00425-006-0474-y

[43] Zentgraf U, Laun T, Miao Y (2010) The complex regulation of WRKY53 during leaf senescence of *Arabidopsis thaliana* . Eur J Cell Biol 89: 133–137. 20004496 10.1016/j.ejcb.2009.10.014

[44] Zhou X, Jiang Y, Yu D (2011) WRKY22 transcription factor mediates dark-induced leaf senescence in Arabidopsis. Mol Cells 31: 303–313. 21359674 10.1007/s10059-011-0047-1 PMC3933965

[45] Guo Y, Cai Z, Gan S (2004) Transcriptome of Arabidopsis leaf senescence. Plant, Cell Environ 27: 521–549.

[46] Fernandez DE, Heck GR, Perry SE, Patterson SE, Bleecker AB, et al (2000) The embryo MADS domain factor AGL15 acts postembryonically. Inhibition of perianth senescence and abscission via constitutive expression. Plant Cell 12: 183–198. 10662856 10.1105/tpc.12.2.183 PMC139757

[47] Fang SC, Fernandez DE (2002) Effect of regulated overexpression of the MADS domain factor AGL15 on flower senescence and fruit maturation. Plant Physiol 130: 78–89. 12226488 10.1104/pp.004721 PMC166541

[48] Hoeberichts FA, van Doorn WG, Vorst O, Hall RD, van Wordragen MF (2007) Sucrose prevents up-regulation of senescence-associated genes in carnation petals. J Exp Bot 58: 2873–2885. 17630294 10.1093/jxb/erm076

[49] Wagstaff C, Yang TJ, Stead AD, Buchanan-Wollaston V, Roberts JA (2009) A molecular and structural characterization of senescing Arabidopsis siliques and comparison of transcriptional profiles with senescing petals and leaves. Plant J 57: 690–705. 18980641 10.1111/j.1365-313X.2008.03722.x

[50] Balazadeh S, Riaño-Pachón DM, Mueller-Roeber B (2008) Transcription factors regulating leaf senescence in Arabidopsis thaliana. Plant Biology 10: 63–75. 18721312 10.1111/j.1438-8677.2008.00088.x

[51] Li J, Dai X, Zhao Y (2006) A role for auxin response factor 19 in auxin and ethylene signaling in Arabidopsis. Plant Physiol 140: 899–908. 16461383 10.1104/pp.105.070987 PMC1400570

[52] Hunter DA, Steele BC, Reid MS (2002) Identification of genes associated with perianth senescence in Daffodil (Narcissus pseudonarcissus L. ‘Dutch Master’). Plant Sci 163: 13–21.

[53] van der Graaff E, Schwacke R, Schneider A, Desimone M, Flugge UI, et al (2006) Transcription analysis of arabidopsis membrane transporters and hormone pathways during developmental and induced leaf senescence. Plant Physiol 141: 776–792. 16603661 10.1104/pp.106.079293 PMC1475451

[54] Breeze E, Wagstaff C, Harrison E, Bramke I, Rogers H, et al (2004) Gene expression patterns to define stages of post-harvest senescence in Alstroemeria petals. Plant Biotechnol J 2: 155–168. 17147607 10.1111/j.1467-7652.2004.00059.x

[55] Xu X, Gookin T, Jiang C-Z, Reid M (2007) Genes associated with opening and senescence of Mirabilis jalapa flowers. J Exp Bot 58: 2193–2201. 17525082 10.1093/jxb/erm058

[56] Saks Y, Staden J (1993) Evidence for the involvement of gibberellins in developmental phenomena associated with carnation flower senescence. Plant Growth Regul 12: 105–110.

[57] Hunter DA, Ferrante A, Vernieri P, Reid MS (2004) Role of abscisic acid in perianth senescence of daffodil (*Narcissus pseudonarcissus*“Dutch Master”). Physiol Plantarum 121: 313–321. 10.1111/j.0031-9317.2004.0311.x 15153199

[58] Porat R, Borochov A, Halevy AH (1993) Enhancement of petunia and dendrobium flower senescence by jasmonic acid methyl ester is via the promotion of ethylene production. Plant Growth Regul 13: 297–301.

[59] Porat R, Reiss N, Atzorn R, Halevy AH, Borochov A (1995) Examination of the possible involvement of lipoxygenase and jasmonates in pollination-induced senescence of Phalaenopsis and Dendrobium orchid flowers. Physiol Plantarum 94: 205–210.

[60] Xu Y, Ishida H, Reisen D, Hanson MR (2006) Upregulation of a tonoplast-localized cytochrome P450 during petal senescence in *Petunia inflata* . BMC Plant Biol 6: 8. 16613603 10.1186/1471-2229-6-8 PMC1540422

[61] Alexander L, Grierson D (2002) Ethylene biosynthesis and action in tomato: a model for climacteric fruit ripening. J Exp Bot 53: 2039–2055. 12324528 10.1093/jxb/erf072

[62] Sugawara H, Shibuya K, Yoshioka T, Hashiba T, Satoh S (2002) Is a cysteine proteinase inhibitor involved in the regulation of petal wilting in senescing carnation (*Dianthus caryophyllus* L.) flowers? J Exp Bot 53: 407–413. 11847238 10.1093/jexbot/53.368.407

[63] Grbić V, Bleecker AB (1995) Ethylene regulates the timing of leaf senescence in Arabidopsis. Plant J 8: 595–602.

[64] Nishiyama K, Guis M, Rose JK, Kubo Y, Bennett KA, et al (2007) Ethylene regulation of fruit softening and cell wall disassembly in Charentais melon. J Exp Bot 58: 1281–1290. 17308329 10.1093/jxb/erl283

[65] van Doorn WG, Balk PA, van Houwelingen AM, Hoeberichts FA, Hall RD, et al (2003) Gene expression during anthesis and senescence in Iris flowers. Plant Mol Biol 53: 845–863. 15082930 10.1023/B:PLAN.0000023670.61059.1d

[66] Hatsugai N, Kuroyanagi M, Nishimura M, Hara-Nishimura I (2006) A cellular suicide strategy of plants: vacuole-mediated cell death. Apoptosis 11: 905–911. 16547592 10.1007/s10495-006-6601-1

