# Supplementary material for: Transcriptome Changes Associated with Delayed Flower Senescence on Transgenic Petunia by Inducing Expression of etr1-1, a Mutant Ethylene Receptor
Source: PLoS One. 2013 Jul 9;8(7):e65800. doi: 10.1371/journal.pone.0065800 (PMC3706537; doi:10.1371/journal.pone.0065800)
Supplement: Table S3 — Gene-specific primers for quantitative real-time PCR. (DOCX) [file pone.0065800.s003.docx]

| Table S3. Gene-specific primers for real-time qRT-PCR | |
| --- | --- |
| Gene name (GeneBank accession number) | PCR primers |
| *PhAOC* (EU652410) | \| F5’-CGGCATTTTTGCAGGAGTTT-3’ \| \| --- \| \| R5’-CCAGCAACTCAGATGGCAGAT-3’ \| |
| *PhERF2* (HQ259596.1) | \| F5’-GCATTTGAATCTGAGATGAAGT-3’ \| \| --- \| \| R5’-ACTTAGTAGACACCTCCCATCA-3’ \| |
| *SAG29* (AF313914.1) | \| F5’-ACGCATTGCAGTCCATGGAGAA-3’ \| \| --- \| \| R5’-CAGTGTCAAGTTCCTTGCCTTACT-3’ \| |
| *HD* (CV299049) | \| F5’-TACAAAGAACCACCACCAGCTC-3’ \| \| --- \| \| R5’-ACTATCTGATCTCTTGACACCCATA-3’ \| |
| *NAC100* (TC1513) | F5’-TCCTGCTCCACCTGCATTTGCTT-3’  R5’-AACCCGGGCTTGCCCAATGA-3’ |
| 26S rRNA | \| F5’-AGCTCGTTTGATTCTGATTTCCAG-3’ \| \| --- \| \| R5’-GATAGGAAGAGCCGACATCGAAGG-3’ \| |
